# Craniofacial changes and symptoms of sleep-disordered breathing in healthy children

**DOI:** 10.1590/2176-9451.20.3.080-087.oar

**Published:** 2015

**Authors:** Maria Christina Thomé Pacheco, Bruna Santos Fiorott, Nathalia Silveira Finck, Maria Teresa Martins de Araújo

**Affiliations:** 1 Full professor, Dental Clinic department, Universidade Federal do Espírito Santo, UFES, Vitória, Brazil; 2 MSc in Dental Clinic, Universidade Federal do Espírito Santo, Vitória, ES. Brazil; 3 Adjunct professor, Physiological Sciences department, Universidade Federal do Espírito Santo, Vitória, ES, Brazil

**Keywords:** Malocclusion, Mouth breathing, Quality of life

## Abstract

**INTRODUCTION:**

The main cause of mouth breathing and sleep-disordered breathing (SDB) in childhood is associated with upper airway narrowing to varying degrees.

**OBJECTIVE:**

The aim of this study was to assess the prevalence of morphological and functional craniofacial changes and the main clinical symptoms of SDB in healthy children.

**METHODS:**

A cross-sectional observational study was conducted. A sample comprising 687 healthy schoolchildren, aged 7-12 years old and attending public schools, was assessed by medical history, clinical medical and dental examination, and respiratory tests. The self-perceived quality of life of mouth breathing children was obtained by a validated questionnaire.

**RESULTS:**

Out of the total sample, 520 children were nose breathers (NB) while 167 (24.3%) were mouth breathers (MB); 32.5% had severe hypertrophy of the palatine tonsils, 18% had a Mallampati score of III or IV, 26.1% had excessive overjet and 17.7% had anterior open bite malocclusion. Among the MB, 53.9% had atresic palate, 35.9% had lip incompetence, 33.5% reported sleepiness during the day, 32.2% often sneezed, 32.2% had a stuffy nose, 19.6% snored, and 9.4% reported having the feeling to stop breathing while asleep. However, the self-perception of their quality of life was considered good.

**CONCLUSION:**

High prevalence of facial changes as well as signs and symptoms of mouth breathing were found among health children, requiring early diagnosis and treatment to reduce the risk of SDB.

## Abstract (pt)

**INTRODUÇÃO:**

a principal causa da respiração bucal e dos distúrbios respiratórios do sono (DRS) está associada ao estreitamento das vias aéreas superiores, em diferentes graus.

**OBJETIVO:**

avaliar a prevalência de alterações morfológicas e funcionais da face e os principais sintomas clínicos de DRS em crianças saudáveis.

**MÉTODOS:**

estudo transversal, observacional, com amostra de 687 escolares saudáveis, provenientes de escolas públicas, com idades entre 7 e 12 anos. Foram avaliados pela história clínica, exame clínico médico e odontológico e testes respiratórios. A autopercepção da qualidade de vida dos escolares com respiração bucal foi obtida por meio de um questionário validado.

**RESULTADOS:**

na amostra total, 520 crianças eram respiradoras nasais (RN) e 167 (24,3%) eram respiradoras bucais (RB); 32,5% tinham hipertrofia das amígdalas palatinas, 18% tinham índice Mallampati obstrutivo (III e IV); 26,1% tinham *overjet* exagerado e 17,7%, mordida aberta anterior. Entre os RB, 53,9% tinham palato atrésico; 35,9% com ausência de selamento labial; 33,5% relataram sonolência diurna; 32,2%, espirros frequentes; 32,2%, nariz entupido; 19,6% roncavam e 9,4% relataram ter a sensação de parar de respirar durante o sono. Entretanto, a autopercepção da qualidade de vida desses escolares foi considerada boa.

**CONCLUSÃO:**

foi encontrada alta prevalência de alterações faciais, de sinais e de sintomas clínicos de respiração bucal nos escolares saudáveis examinados, necessitando diagnóstico e tratamento para reduzir o risco de DRS.

## INTRODUCTION

Sleep-disordered breathing (SDB) varies in severity; ranging from snoring, upper airway resistance syndrome (UARS) and, the most severe, obstructive sleep apnea (OSA). In contrast with SDB in adults, which is usually associated with obesity, the pediatric population experiences these disorders in association with hypertrophy of tonsils and adenoids, allergies, frequent colds and mouth breathing.^1^^-^ 7

The association between mouth breathing and SDB has been described as a public health concern because of the clinical impact of these disorders on craniofacial development and growth, delay in height and weight growth and behavioral changes, such as hyperactivity, irritability, restless sleep, impaired concentration and reduced school performance.^8^^,^ 9 ^,^ 10

SDB during childhood is rather frequent. However, its signs are not always appropriately recognized or even diagnosed. Anamnesis should tackle aspects of sleep pattern, especially in mouth-breathing children.^2^^-^ 5

With regards to the impact on craniofacial growth and development, the persistence of mouth breathing throughout the growth stage causes specific facial changes, such as vertical increase of the lower face; narrow palate; dental malocclusions, mainly anterior open bite and posterior crossbite; lip incompetence; short upper lip and everted lower lip; hypotonic masticatory muscles; and changes in tongue posture at resting, swallowing, speaking and chewing.^8^^-^ 11

Because mouth breathing is one of the predisposing factors to SDB in children, this study aims to assess the prevalence of the morphological and functional craniofacial changes and the main nasal and sleep symptoms reported by healthy schoolchildren aged between 7 and 12 years old.

## MATERIAL AND METHODS

A cross-sectional, observational study with a quantitative epidemiological approach was conducted with 687 schoolchildren aged between 7 and 12 years old. The subjects were from eight different elementary schools, each school randomly selected from each one of the eight micro regions of the city Vitória, ES, Brazil. The selection of healthy schoolchildren, rather than patients seeking medical or dental care, was due to lack of a preventive policy for health problems in Brazilian public schools. Moreover, most parents or legal guardians only seek health care when the child already has some type of disease symptom.

All schoolchildren were invited to participate; however, we examined only those whose parents or legal guardians had signed the informed consent form (ICF) allowing them to participate. Exclusion criteria were as follows: unerupted first molars, previous orthodontic treatment, patients under medical care and presenting some type of neurological, neuromuscular or motor changes that would hinder their participation.

This study was approved by Universidade Federal do Espírito Santo Institutional Review Board and had permission from local City Hall to be conducted in the public schools.

The examiners (two orthodontists and two Otolaryngology internship medical residents) underwent calibration. Average values for inter- and intraexaminer agreement of 0.84 and 0.93 were obtained by *Kappa* test.

A survey instrument was designed to collect the main morphological and functional clinical characteristics of the face, occlusion and upper airways. In order to have some of their facial features assessed, children were carefully observed in their natural state, without letting them realize that they were being examined. Each child was assessed under natural light, sitting on a chair in front of the researcher who wore biosafety equipment and disposable material for clinic examination.

The medical residents examined facial and airway features (seeking for size, coloration, nasal secretions, obstruction and edema). Dental students examined craniofacial features, malocclusion and performed the respiratory tests. The mirror test was performed by placing a graded mirror under the nose, and a halo of water vapor was marked after the third normal expiration. Two lip seal tests were also performed for three minutes each, one using a sticky tape to close the lips and the other with water into the child's mouth. At the end of the clinical examination, a diagnostic impression was established by both medical and dental groups, concerning the child's respiratory function. Each child was classified as mouth breather (MB) or nose-breather (NB), distinguishing between chronic and occasional mouth breathers.

Only children diagnosed as mouth breathers answered a structured questionnaire titled "The mouth breather quality of life questionnaire", developed^11^ and validated^12^ by Ribeiro.^11^^,^ 12 The questionnaire comprises 51 questions divided into seven fields to assess mouth breathers' quality of life in terms of nasal problems, sleeping problems, eating disorders, dentition and esthetics, education, communication and atopy. An ordinal value was associated to the sequential answer scale: 0 (zero) for "no/never", 1 or 2 for "hardly ever", 3 for "once in a while", 4 or 5 for "often", 6 for "always", with the highest score referring to the worst quality of life. However, in the present study, we focused mainly on the fields "nasal problems" and "sleeping problems" to assess the schoolchildren self-perception on some of the main symptoms of SDB during childhood.

## Statistical analysis

Statistical tests for comparison of two proportions were performed considering two populations with a Bernoulli distribution and parameters *p*_1_ and *p*_2_. For larger samples, the proportions were considered an approximation of the normal distribution *N* (0.1). It was assumed that the null hypothesis was *p*_1 _= *p*_2_. An alpha level of 5% and a confidence interval of 95% were adopted. The statistical programs used were Action (with the system developed under the R platform) and IBM SPSS Statistics 19.

## RESULTS

The initial sample comprised 687 healthy schoolchildren of which 329 (47.9%) were males and 358 (52.1%) were females. The majority of schoolchildren aged between 8 and 9 years old (42.9%). The distribution per school varied according to the size of the school and the survey acceptance through the signing of an ICF by the parent/legal guardian. From the total sample, 167 (24.3%) schoolchildren were diagnosed as MB while 520 (75.7%) were diagnosed as NB.

Table 1 shows the main alterations on the upper airways (UA). The occurrence of palatine tonsils (grades III and IV) and Mallampati classes II, III and IV were dominant in MB schoolchildren. These data are relevant and point to the predominance of narrowed airways in MB schoolchildren.

**Table 1.** Prevalence of upper airway (UA) problems in nose breathers and mouth breathers schoolchildren.

	Groups				p value	Total	
	Nose breathers		Mouth breathers			n	%
	n	%	n	%			
Palatine tonsils							
I	83	16.0	20	12.0	0.230	103	15.0
II	281	54.0	80	47.9	0.167	361	52.5
III	138	26.5	59	35.3	0.025*	197	28.7
IV	18	3.5	8	4.8	0.434	26	3.8
Mallampati score							
I	221	42.5	41	24.5	0.000*	262	38.2
II	219	42.1	82	49.1	0.113	301	43.8
III	71	13.7	37	22.2	0.009*	108	15.7
IV	9	1.7	7	4.2	0.067	16	2.3
Nasal septum							
Normal	302	58.1	67	40.1	0.000*	369	53.7
Swollen	119	22.9	61	36.5	0.000*	180	26.2
Deviated	86	16.5	32	19.2	0.492	118	17.2
Swollen/ deviated	13	2.5	7	4.2	0.383	20	2.9
Turbinate hypertrophy							
No	223	42.9	45	26.9	0.000*	268	39.0
Yes	297	57.1	122	73.1	0.000*	419	61.0

* Statistically significant (p < 0.05).

Changes in nasal septum and turbinate hypertrophy were also more prevalent in the MB group, highlighting edema (36.5%), nasal septal deviation (19.2%) and turbinate hypertrophy (73.1%). Some children simultaneously experienced edema and deviated nasal septum. The presence of tonsils grade III, Mallampati class III, edema of the nasal septum and turbinate hypertrophy were relevant findings for the MB group (p < 0.050).

Table 2 describes the most important facial features assessed. Although most schoolchildren have a normal facial type (mesofacial), there is a high incidence of the dolichofacial pattern (34.7%) in the MB group. This pattern was perceived in only 11.7% of the NB group. Convex facial profile was predominant (50.9%) in the MB group. Absence of lip competence was higher in the MB group (35.9%) in comparison to the NB group in which it was only 2.5%.

**Table 2.** Prevalence of facial features in nose breathers and mouth breathers schoolchildren.

	Groups				p value	Total	
	Nose breathers		Mouth breathers			n	%
	n	%	n	%			
Facial type							
Mesofacial	404	77.7	100	60.5	0.000*	505	73.5
Dolichofacial	61	11.7	58	34.7	0.000*	119	17.3
Brachyfacial	55	10.6	8	4.8	0.000*	63	9.2
Facial profile							
Straight	367	70.6	75	44.9	0.000*	442	64.3
Convex	142	27.3	85	50.9	0.000*	227	33.1
Concave	11	2.1	7	4.2	0.144	18	2.6
Lip competence							
Present	507	97.5	107	64.1	0.000*	614	89.3
Absent	13	2.5	60	35.9	0.000*	73	10.7

* Statistically significant (p < 0.05).

The most relevant findings in the MB group (p < 0.050) were dolichofacial pattern and the absence of lip competence.

Table 3 shows the prevalence of malocclusion in schoolchildren. The MB group presented a prevalence of deep overbite, anterior open bite, posterior crossbite, pronounced overjet, Angle Class II molar relationship and atresic palate. The relevant findings (p < 0.050) were overjet greater than 4 mm and atresic palate.

**Table 3.** Prevalence of malocclusion in nose breathers and mouth breathers groups

	Groups				p value	Total	
	Nose breathers		Mouth breathers			n	%
	n	%	n	%			
Overbite							
Normal	271	52.1	73	43.7	0.072	344	50.1
Moderate	135	26.0	45	26.9	0.897	180	26.2
Severe	24	4.6	9	5.4	0.832	33	4.8
Does not apply	90	17.3	40	24.0	0.070	130	18.9
Open bite							
Absent	437	84.1	128	76.6	0.025*	565	82.3
Top	40	7.7	18	10.8	0.274	58	8.4
Present	43	8.2	21	12.6	0.121	64	9.3
Crossbite							
Absent	417	80.2	137	82.0	0.909	554	80.6
Anterior	28	5.4	4	2.4	0.165	32	4.7
Posterior	67	12.8	24	14.4	0.689	91	13.2
Ant/Posterior	8	1.6	2	1.2	0.997	10	1.5
Overjet							
1-2 mm	341	65.6	88	52.7	0.004*	429	62.4
3-4 mm	109	21.0	48	28.7	0.051	157	22.9
> 4 mm	11	2.1	11	6.6	0.009*	22	3.2
Does not apply	59	11.4	20	12	0.943	79	11.5
Molar relationship							
Class I	409	78.7	123	73.7	0.098	532	77.5
Class II	92	17.7	35	21.0	0.400	127	18.5
Class III	19	3.7	9	5.4	0.460	28	4.1
Palate							
Normal	328	63.1	77	46.1	0.000*	405	58.9
Atresic	192	36.9	90	53.9	0.000*	282	41.0
TOTAL	520	100.0	167	100.0		687	100.0

* Statistically significant (p < 0.05).

On the mirror test (Table 4), most children (95.3%) showed a halo of water vapor larger than 30 mm, which demonstrates lack of nasal obstruction. However, when assessing the MB group, the prevalence of a halo smaller than 30 mm (14.4%) was statistically significant, which suggests that the presence of mouth breathing was caused by nasal obstruction.

**Table 4.** Prevalence found for respiratory tests in nose breathers and mouth breathers schoolchildren.

	Groups				p value	Total	
	Nose breathers		Mouth breathers			n	%
	n	%	n	%			
Mirror test							
Halo > 30 mm	512	98.5	143	85.6	0.000*	655	95.3
Halo < 30 mm	8	1.5	24	14.4	0.000*	32	4.7
Lip seal test							
For 3 minutes	510	98.1	86	51.5	0.000*	596	86.8
Less than 3 minutes	10	1.9	81	48.5	0.000*	91	13.2
Water retention test							
For 3 minutes	511	98.3	90	53.9	0.000*	601	87.5
Less than 3 minutes	9	1.8	77	46.1	0.000*	86	12.5
TOTAL	520	100.0	167	100.0		687	100.0

* Statistically significant (p < 0.05).

On both lip seal tests, using sticky tape (48.5%) or water retention (46.1%), the prevalence of lip seal for less than 3 minutes was significant for the MB group, which suggests difficulty breathing through the nose.

The results yielded from the quality of life questionnaire applied to mouth breathers are shown in Table 5. They show the seven fields surveyed and the overall score. Higher scores mean worse quality of life, while lower scores show better quality of life. Both mean and median values in all fields were below half the maximum value possible for each field, showing good assessment of quality of life. However, taking into account the wide variability of the standard deviation, one can see that a great part of children reported having a not so good quality of life.

**Table 5.** Median, mean and overall score obtained with the quality of life questionnaire for mouth breathers at the seven assessed fields.

Fields	Lowest value	Highest value	Median	Mean	Standard deviation
Nose problem	0	52	17.00	18.08	10.73
Sleeping problem	0	51	19.00	20.34	11.95
Food	0	35	15.00	13.98	7.98
Dentition / esthetic	0	26	10.00	10.29	6.27
Education	0	18	6.00	5.99	4.42
Communication	0	47	21.00	18.51	11.69
Atopy	0	48	15.00	16.41	11.96
Overall score	0	241	102.00	103.61	48.07

**Table 6.** Prevalence of the main questions related to the nose-problems and sleep-problems fields reported by the MB group (adapted from Ribeiro11).

Nose problems field	Frequency (%)	Sleep problems field	Frequency (%)
Do you have any nasal problems?	32.00	Do you have any sleeping problems?	33.54
Does your nose bother you?	30.00	Is your sleep usually restless?	31.00
Do you often feel a stuffy nose?	32.27	Do you wake up during the night?	32.27
Do you often sneeze?	32.27	Do you often complain of being sleepy during the day?	33.54
Do you present any nose itching?	22.78	Do you sleep with your mouth open?	38.60
Do you ever have a runny nose?	17.72	Have you ever stopped breathing while sleeping?	9.49
Do you snore at night?	19.62	Do you ever wake up with a headache?	11.39
Do you ever feel your mouth or throat itch?	23.41	Does your mouth feel dry when you wake up?	41.13

## DISCUSSION

The presence of hypertrophied palatine tonsils was remarkable for the entire sample, with predominance of grades I and II (non obstructive) in the NB group and grades III and IV (obstructive) in the MB group. Palatine tonsils were classified according to Brodsky:^13^ grade I (tonsils take up 25% of the oropharyngeal airway space), grade II (25 to 50%), grade III (50 to 75%) and grade IV (more than 75% of the oropharyngeal airway space).

The American Academy of Pediatrics^1^ highlights hypertrophy of palatine tonsils as a significant risk factor for the development of SDB during childhood. Nevertheless, the severity of SDB is not always related to the size of tonsils. Many children with significant hypertrophy do not present breathing disorders related to sleep. Therefore, other risk factors, such as changes in facial morphology or alterations in breathing control during sleep, may coexist. For this reason, we surveyed most morphological, craniofacial and upper airway changes related to SDB in children.

Unlike tonsils grades that identify lateral airway narrowing, the Mallampati score identifies vertical airway narrowing. A modified Mallampati score,^14^^,^ 15 in total mouth opening, with the tongue relaxed and lying in the oral cavity, was used. In the NB group, there was a predominance of class I Mallampati, which suggests wide airways. In the MB group, Mallampati classes III and IV (obstructive) were predominant, which suggests narrow or small airways.^6^^,^ 7 The presence of a swollen or deviated nasal septum (59.9%) in the MB group was striking, as it contributes to increased mouth breathing. Palombin et al^3^ recommend *investigating any type of nasal obstruction, turbinate hypertrophy, Mallampati score, size of palatine tonsils, long face, genetic syndrome patterns and mouth breathing during clinical examination of children with suspected SDB. In the present study, long face pattern (dolichofacial), convex profile and absence of lip seal were predominant in MB children.*

The main morphological changes found were: atresic palate, anterior open bite, posterior crossbite and excessive overjet. These malocclusions are mainly caused by an imbalance between forces exerted by the tongue, lips and the perioral muscles.^16^ *,* 17 In the MB group, atresic palate and overjet equal to or greater than 3 mm were more prevalent. A V-shaped palatal arch, with maxillary width equal or smaller than the mandible, was classified as an atresic palate. Some schoolchildren showed several concurrent morphological changes.

The mirror test^18^,^19^,^20^ helped to detect the presence of upper airway obstruction and the predominant breathing pattern (whether nose-breather or mouth breather). However, the water retention tests were important in determining the differential diagnosis between obstructive mouth breathing and an acquired habit of mouth breathing after temporary obstruction. The consequences associated with morphological changes will be the same, but knowing the cause for mouth breathing (whether obstruction or habit) provides the most effective treatment for the child. 

The lip seal test (51.5%) and the water retention test (53.9%) for 3 minutes in the MB group revealed there was a slight predominance of mouth breathing habits in comparison to obstructive mouth breathing. 

Only *the MB group completed "The mouth breather quality of life questionnaire" to determine schoolchildren self-perception of their quality of life, *as recommended by Ribeiro.^11^ The authors of the present study tried to encourage the participation of children, believing that there is information that only they could provide and thus be closer to their reality. The American Academy of Pediatrics^1^ and other pediatric organizations recommend the involvement of children and direct questioning about their health conditions and functions, mainly for those over the age of 6.^21^

*According to the questionnaire, 54% of schoolchildren quality of life self-perception was considered good, with below-average scores and mostly negative answers. This result is not surprising and can be supported by school attendance records and children's good health. However, 46% of those children had a negative quality of life self-perception. The most expressive results were those related to sleeping problems and nose-related problems. These schoolchildren reported having the feeling to stop breathing while asleep, waking up with headache, having daytime sleepiness, sleeping with an open mouth, waking up with dry mouth, runny nose, snore, stuffy nose and sneezing. According to Palombini,* 2 *Palombini et al,* 3 *and *Guilleminault* et al,* 4 *these are some of the major symptoms of SDB during childhood.*

Popoaski et al^22^ also applied a quality of life questionnaire adapted from Ribeiro.^11^ They found that sleep-problem and nose-problem fields were the ones with greater scoring, thereby suggesting that these fields can negatively affect the quality of life of mouth breathers. In their study,^22^ the prevalence of trouble while asleep was nearly three times greater than in the study by Ribeiro.^11^

Although most schoolchildren reported having good self-perception of their quality of life, the prevalence of signs and symptoms related to SDB during childhood was high in the present study. According to Zettergren-Wijk et al,^23^ SDB in young children has an unfavorable effect on the development of several dental and facial components. However, if SDB is diagnosed and treated at an early age, nearly complete normalization of dentofacial morphology may be achieved. 

*Due to the negative repercussion of mouth breathing and its close relation to functional and morphological facial changes that favour SDB during childhood, the implementation of policies to prevent breathing problems is of particular relevance, as clearly evidenced by our results.* These preventive measures must be set to provide proper nasal breathing via orthodontic and otolaryngological treatment, so as to develop educational guidance strategies and to stimulate healthy habits that might avoid mouth breathing. Furthermore, signs and symptoms related to SDB in children should be carefully investigated to justify the need for referrals to specific diagnostic exams.

## CONCLUSION

» The prevalence of functional and morphological facial changes was considerably high among all schoolchildren assessed, mainly in those diagnosed as mouth breathers (MB).

» The most prevalent alterations found in MB, in order of prevalence, were: deviated or swollen nasal septum; atresic palate; hypertrophic tonsils; lip incompetence; dolichofacial pattern; excessive overjet; anterior open bite; Mallampati classes III and IV; and posterior crossbite.

» Self-perception of children's quality of life was considered good for most MB schoolchildren despite the high prevalence of SDB symptoms reported, mainly those related to nasal and sleep problems.
